# External quality assurance of molecular serotype detection of dengue virus RNA among virus research and diagnostic laboratories in India

**DOI:** 10.3389/fcimb.2026.1919409

**Published:** 2026-09-03

**Authors:** Gopinath Ramalingam, Selvi Krishnan, Labanya Mukhopadhyay, Nivedita Gupta, Madhumitha Patchaiyappan, Krishnapriya Subramani, Harmanmeet Kaur, Sucila Thangam Ganesan

**Affiliations:** 1 Virus Research and Diagnostic Laboratory, Department of Microbiology, Government Theni Medical College, Theni, India; 2 Indian Council of Medical Research, V. Ramalingaswami Bhawan, Ansari Nagar, New Delhi, India; 3 Scientist G & Head, Communicable Diseases, Indian Council of Medical Research, V. Ramalingaswami Bhawan, New Delhi, India

**Keywords:** dengue virus, external quality assessment scheme, molecular serotyping, proficiency testing, real-time RT-PCR, VRDL

## Abstract

**Background:**

External Quality Assessment Schemes (EQAS) are essential for ensuring the accuracy, reliability, and comparability of molecular diagnostic testing across laboratory networks. To strengthen dengue molecular surveillance and laboratory quality systems in India, a Pan-Dengue Molecular Serotyping EQAS was developed and implemented among Virus Research and Diagnostic Laboratories (VRDLs) in India.

**Methods:**

A nationwide EQAS was conducted in three rounds between December 2024 and February 2026. Proficiency testing panels consisting of five coded lyophilized serum samples (500 µL each) containing dengue-positive and dengue-negative specimens were prepared, characterized, validated, and distributed to participating laboratories. Homogeneity and stability assessments were performed prior to distribution. Participating laboratories tested the panels using their routine molecular diagnostic workflows and reported dengue detection and serotype identification results through the National Institute of Epidemiology (NIE) PT Panel Portal. Laboratory performance was evaluated based on concordance with assigned reference results.

**Results:**

A total of 34, 40, and 56 laboratories enrolled in Round 1, Round 1 Phase-II, and Round 2, respectively. The corresponding response rates were 91.2%, 95.0%, and 94.6%. Complete concordance was achieved by all reporting laboratories in Round 1 (31/31) and Round 1 Phase-II (38/38). In Round 2, 50/53 laboratories (94.3%) achieved 100% concordance, while two and one laboratory achieved 80% and 60% concordance, respectively. The overall pass rate across all rounds was 97.5%.

**Conclusion:**

The Pan-Dengue Molecular Serotyping EQAS highlighted a high level of proficiency among participating laboratories and effectively strengthened molecular diagnostic quality assurance within the national laboratory network. Regular implementation of such programmes will support reliable dengue surveillance, continuous quality improvement, and enhanced preparedness for dengue outbreaks in India.

## Introduction

Dengue remains one of the most important viral diseases under public health surveillance in India, requiring timely and accurate laboratory diagnosis for effective disease monitoring, outbreak investigation, and implementation of control measures (Gupta et al., 2025; Sanap et al., 2026; Shinde et al., 2026). Molecular characterization of dengue virus (DENV) is essential for surveillance, outbreak investigations, and monitoring of circulating viral strains. The four antigenically distinct serotypes (DENV-1, DENV-2, DENV-3, and DENV-4) exhibit differences in geographical distribution, epidemic potential, and association with disease severity (Verma et al., 2023; Umar et al., 2025). Continuous monitoring of circulating serotypes supports understanding of transmission patterns, detection of serotype shifts, identification of emerging strains, and implementation of appropriate public health interventions (Songjaeng et al., 2022). Real-time reverse transcription polymerase chain reaction (RT-PCR) has become a preferred molecular diagnostic method for early dengue detection due to its high sensitivity, specificity, and ability to detect viral RNA during the acute phase of infection (Nunes et al., 2021). The availability of molecular diagnostic platforms has significantly enhanced the capacity of public health laboratories to generate rapid and reliable results, strengthening dengue surveillance and outbreak response activities (Liu et al., 2023). In India, the Department of Health Research (DHR), Government of India, established the Virus Research and Diagnostic Laboratory (VRDL) network to improve diagnostic infrastructure for viral diseases. The network comprises laboratories established in medical colleges, regional centres, and public health institutions, providing diagnostic support for routine surveillance and outbreak investigations (Das et al., 2024). With the expansion and decentralization of molecular testing services, maintaining accuracy, consistency, and comparability of results generated by geographically distributed laboratories remains a major challenge. Differences in specimen processing, nucleic acid extraction methods, assay performance, reagent quality, equipment maintenance, and technical expertise may influence laboratory outcomes and affect diagnostic reliability (Anvari et al., 2020; Alagarasu et al., 2021; Starolis, 2025). Therefore, implementation of robust quality assurance systems is essential to maintain confidence in molecular diagnostic services.

External Quality Assessment Schemes (EQAS) provide an independent evaluation of laboratory performance by comparing participant results with reference standards and peer laboratories (Picconi et al., 2021; Johnson, 2024; Adekoya et al., 2025; Chen et al., 2025; Christudoss and Amirtharaj, 2026). EQAS programmes are recognized as important tools for assessing analytical competency, identifying testing limitations, promoting standardization, and supporting continuous quality improvement (Montalvão et al., 2022; Buchta et al., 2025). This is particularly important for molecular assays, where variations in sample quality, extraction efficiency, amplification performance, contamination control, and result interpretation may affect diagnostic accuracy (Laudus et al., 2022; Benschop et al., 2024). Participation in proficiency testing enables laboratories to evaluate their testing performance, implement corrective actions, and strengthen quality management practices (Ricós et al., 2022; Abegaz et al., 2023; Kostyanev et al., 2025).

Globally, dengue EQAS programmes have primarily focused on evaluating serological detection (NS1 antigen and IgM/IgG) and molecular detection of dengue virus RNA, with limited emphasis on assessing serotype identification competency. In India, existing quality assurance initiatives have largely addressed routine dengue diagnosis, while a structured nationwide proficiency testing programme for molecular serotyping has not been established. Molecular serotyping presents additional challenges due to the need for accurate discrimination of DENV-1 to DENV-4, influenced by viral genetic diversity, assay performance, and technical expertise (Phadungsombat et al., 2024). The present EQAS addresses this critical gap by evaluating laboratory proficiency in both dengue virus detection and serotype identification, thereby strengthening molecular surveillance and diagnostic quality systems (Araujo et al., 2026; Thuy et al., 2026).

The programme utilized well-characterized serum-based PT materials containing dengue viral RNA, representing different serotypes, which were validated for homogeneity, stability, and performance before distribution. The present study describes the development, validation, distribution, and evaluation of a nationwide Pan-Dengue Molecular Serotyping EQAS conducted among VRDL laboratories across India between December 2024 and February 2026. The study assesses laboratory performance, concordance of dengue virus detection and serotyping, and the role of EQAS in strengthening molecular diagnostic capacity, laboratory quality systems, and national dengue surveillance.

## Materials and methods

### Study design

A nationwide External Quality Assessment Scheme (EQAS) for molecular detection and serotyping of dengue virus was implemented by the VRDL, Government Theni Medical College, Theni, Tamil Nadu, India under the Indian Council of Medical Research (ICMR) scheme “To enhance the quality of infectious disease diagnosis in India through implementation of an External Quality Assurance Program”. The programme was conducted in multiple rounds between December 2024 and February 2026 to evaluate the proficiency of participating laboratories in dengue virus detection and serotype identification using routine molecular diagnostic methods. The entire workflow of the EQAS program has illustrated as road map in **Supplementary Figure S1**.

The EQAS was conducted in three phases: Round 1 (December 2024), Round 1 Phase-II (July 2025), and Round 2 (February 2026). Laboratories belonging to the VRDL network across India were invited to participate through official communication. Institutional Ethics Committee approval was obtained for the use of archived and characterized serum samples utilized for panel preparation (Ref. No. GTMC/IEC/2026/29 dated 29/01/2026).

### Participating laboratories

Participating laboratories were enrolled voluntarily following circulation of enrollment forms (Google form). Administrative information includes laboratory details, contact information, testing methodology, molecular platforms, extraction methods, and kit details were collected from each participating centre. A total of 34 laboratories participated during Round 1, 40 laboratories during Round 1 Phase-II, and 56 laboratories during Round 2.

### Selection of samples

Clinical serum specimens positive for dengue virus were selected from archived samples collected through routine dengue surveillance activities of the Virus Research and Diagnostic Laboratory (VRDL) network during [April 2024 to January 2026]. The samples collected from patients with suspected dengue infection within one week of illness onset were initially screened for dengue NS1 antigen and/or dengue IgM positivity and subsequently confirmed for dengue virus RNA by real-time RT-PCR. The selected specimens were stored at -80 °C under controlled conditions until panel preparation.

### Inclusion criteria

Samples were included based on confirmed dengue virus RNA positivity, availability of adequate sample volume, acceptable RNA quality, and confirmed dengue virus serotype identity. Specimens representing different dengue virus serotypes (DENV-1, DENV-2, DENV-3, and DENV-4) with variable viral RNA concentrations and Ct values were selected to assess laboratory proficiency in dengue virus detection and molecular serotyping.

### Exclusion criteria

Samples with insufficient volume, poor RNA quality, inconclusive molecular results, evidence of repeated freeze-thaw cycles, or positivity for HIV, HBV, and HCV infections were excluded from the EQAS panel. Specimens that did not meet the predefined acceptance criteria for molecular characterization, homogeneity, or stability assessment were also excluded prior to final panel preparation and distribution.

### Preparation of proficiency testing panels

A repository of dengue-positive and dengue-negative serum samples was maintained for the preparation of PT panels. Dengue virus serotype confirmation of the proficiency testing (PT) panels was performed at the coordinating reference laboratory using two validated real-time RT-PCR assays: the RealStar^®^ Dengue Type RT-PCR Kit 1.0 RUO (Altona Diagnostics GmbH, Hamburg, Germany), the QuantiPlus™ Dengue Virus Serotyping Real-Time RT-PCR Kit (Huwel Lifesciences Pvt. Ltd., Hyderabad, India), and the TRUPCR^®^ Dengue Typing Kit (3B BlackBio Biotech India Ltd., Bhopal, India). All assays were carried out using calibrated real-time PCR instruments, including Applied Biosystems 7500 Real-Time PCR System (Thermo Fisher Scientific, USA) and Bio-Rad CFX96 Touch Real-Time PCR Detection System (Bio-Rad Laboratories, USA). Both platforms are 96-well, five-channel fluorescence-based real-time PCR systems capable of multiplex detection using hydrolysis probe-based assays. Amplification was performed according to the manufacturer’s recommended cycling conditions, and fluorescence signals were acquired during each amplification cycle for qualitative detection and serotype identification of dengue virus. The serotyping results obtained using these assays were used as the reference results for PT panel characterization and evaluation of participating laboratories.

The characterized serum samples were aliquoted and lyophilized under controlled laboratory conditions. Each vial contained 500 µL of lyophilized serum sample. Serum samples selected for PT panel preparation were aliquoted into sterile cryovials and subjected to lyophilization using an ANM Industries freeze dryer (Model FD-50, India; operating voltage: 220 V). The serum aliquots were initially frozen at −80 °C and subsequently subjected to a controlled freeze-drying cycle comprising freezing, primary drying, and secondary drying phases. During the primary drying phase, the chamber temperature was maintained at −60 °C, and the vacuum pressure was controlled at <100 mTorr using the integrated vacuum gauge (vacuometer) of the freeze dryer to facilitate efficient sublimation of ice crystals. The secondary drying phase was performed at +20 °C for 4–6 h under reduced pressure to remove residual bound moisture. No additional stabilizers or preservatives were incorporated during PT panel preparation. The prepared lyophilized PT materials were further evaluated for reconstitution characteristics, homogeneity, and stability before dispatch.

The PT panel consisted of five coded samples containing dengue-positive (one or more serotypes) and dengue-negative specimens. The identity and expected results of the samples were known only to the organizing laboratory to ensure blinded evaluation.

### Panel characterization and validation

Prior to distribution, all PT samples underwent comprehensive characterization, homogeneity assessment, stability evaluation, and validation. Source samples were initially characterized using dengue real-time RT-PCR and molecular serotyping assays, and positive samples were confirmed by two independent reference methods before inclusion in the PT panel. To assess homogeneity, 10% of aliquots from each batch were randomly selected and tested using the reference molecular assay to ensure uniform distribution of viral RNA and consistency of serotype assignment. Stability studies were performed under different storage conditions, including −20 °C, 2-8 °C, and room temperature, and samples were tested at predetermined intervals to evaluate the stability of viral RNA and serotype detection during storage and transportation. Following aliquoting and lyophilization, representative PT panels were validated using the reference assays. Samples were considered suitable for distribution only when the expected results were consistently obtained and all assay quality control and acceptance criteria were fulfilled.

### Distribution of PT panels

The lyophilized PT panels were distributed to participating laboratories using triple-layer packaging in accordance with International Air Transport Association (IATA) guidelines for transportation of infectious substances. The validated stability of the lyophilized samples enabled transportation at ambient temperature (25 ± 5 °C) during shipment. Upon receipt, participating laboratories were instructed to store the PT panels at 2-8 °C, as recommended in the accompanying instructions, until reconstitution and testing. Following reconstitution of the lyophilized serum samples, laboratories were instructed to store the reconstituted PT materials at -20 °C until testing to maintain sample integrity.

Each shipment included:

- PT panel samples
- Instructions for sample reconstitution
- Sample storage guidelines
- Result reporting format
- Timeline for submission of results

Participating laboratories were instructed to reconstitute the lyophilized serum samples prior to testing according to the instructions provided.

### Testing by participating laboratories

Participating laboratories processed the PT samples using their routine workflow for dengue molecular diagnosis. RNA extraction, amplification, detection, and serotyping were performed using the laboratories’ routine extraction methods, real-time RT-PCR kits, and instrument platforms.

The laboratories were requested to report:

- Sample code
- Dengue detection result (Positive/Negative)
- Dengue virus serotype identified (DENV-1, DENV-2, DENV-3, or DENV-4)
- Cycle threshold (Ct) value
- Extraction method used
- RT-PCR kit details
- Instrument platform used

Results were submitted electronically within 15 working days of receipt of the PT panels.

### Data collection and reporting

Participating laboratories were instructed to analyze the proficiency testing (PT) panel samples using their routine molecular testing workflow and submit the results within 15 working days of receipt of the panels. The laboratories entered their results, including dengue virus detection status, serotype identification, Ct values, assay details, and testing platform information, through the National Institute of Epidemiology (NIE) Portal under the designated PT Panel section.

All submitted data were reviewed and compiled by the organizing laboratory. Reference results, participant results, Ct values, kit details, and performance indicators were entered and maintained in the NIE PT Panel Portal database for analysis. The portal facilitated centralized data management, performance evaluation, concordance analysis, report generation, and communication of individual laboratory feedback. Laboratory performance was assessed by comparing the participant-reported results with the assigned reference results for dengue virus detection and serotype identification.

The assigned reference targets for each PT panel were established during pre-distribution validation and included dengue virus detection status, corresponding serotype identification, and reference Ct values. Since Ct values may vary between molecular platforms and assay systems, an acceptable variation of ±2 Ct from the assigned reference Ct value was considered during evaluation. However, the laboratory performance was determined based on concordance with the dengue detection status and correct serotype identification, while reported Ct values were used as supportive analytical parameters.

### Data analysis and statistical evaluation

Data analysis was performed in accordance with the statistical principles described in ISO 13528:2022; statistical methods for use in proficiency testing by inter laboratory comparison. The real-time RT-PCR cycle threshold (Ct) values were used as the quantitative measurement parameter for evaluation of the dengue molecular serotyping proficiency testing (PT) materials. The between-sample standard deviation (Ss) was calculated and compared with the predefined acceptance criterion of Ss ≤ 0.3 × SDPA, where SDPA represents the standard deviation for proficiency assessment. PT materials were considered homogeneous when the calculated variability among vials remained within the specified acceptance limit.

Stability of the PT materials was evaluated by comparing the mean Ct values obtained under different storage and transportation conditions with the baseline mean Ct value assigned immediately after preparation. The absolute difference between the mean Ct values of stored and reference samples [ABS (x̄ − ȳ)] was calculated. Stability was considered acceptable when the observed difference remained within the predefined acceptance criterion of ABS (x̄ − ȳ) ≤ 0.3 × SDPA. For evaluation of EQAS performance, participant laboratory results were compared with the assigned reference results established during PT panel validation. Laboratory proficiency was assessed based on the accuracy of dengue virus detection (positive/negative agreement) and correct identification of dengue virus serotypes (DENV-1, DENV-2, DENV-3, and DENV-4). The overall performance of participating laboratories was expressed as the percentage of concordant results with the assigned values. All statistical calculations and data analysis were performed using Microsoft Excel (Microsoft Corporation, Redmond, WA, USA). As the evaluation criteria were predefined according to ISO 13528 recommendations for proficiency testing, inferential statistical tests and significance analyses were not applied.

### Corrective actions

Laboratories reporting discordant or aberrant results were contacted individually for troubleshooting and root-cause analysis. Potential causes of error, including extraction failure, assay performance issues, contamination, instrument-related problems, and reporting inaccuracies, were investigated. Appropriate corrective and preventive actions (CAPA) were recommended, and repeat testing was advised whenever necessary.

### Feedback and reporting

Following completion of each EQAS round, individual evaluation reports were generated and communicated to all participating laboratories. The reports included expected results, laboratory performance, concordance analysis, and recommendations for quality improvement. Consolidated performance reports were prepared to assess the overall effectiveness of the Pan-Dengue Molecular Serotyping EQAS programme and to support continuous strengthening of molecular diagnostic quality assurance within the VRDL network.

## Results

### Participation of laboratories

**Figure 1:**
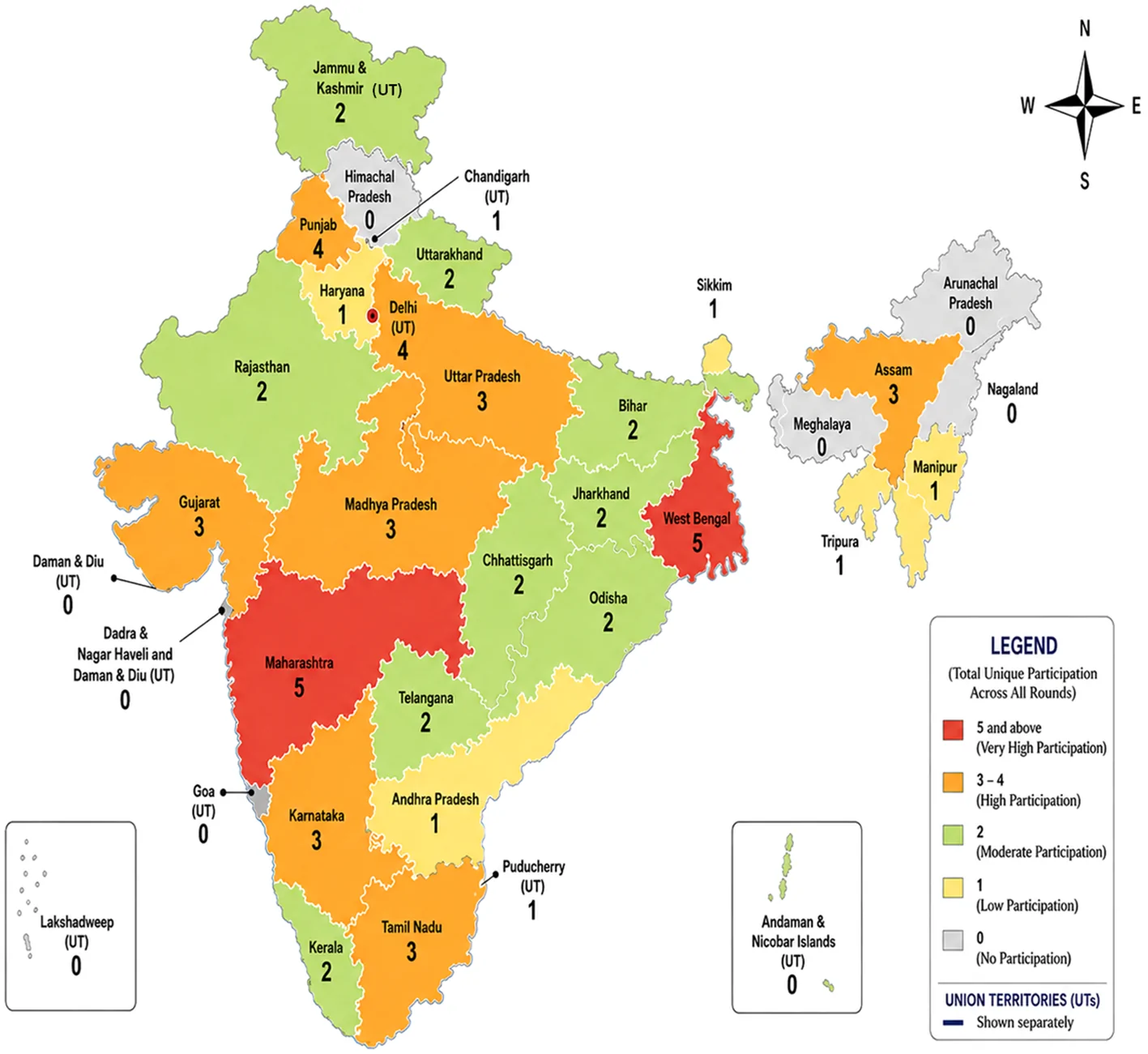
Distribution of participating laboratories in different EQAS rounds across India.

A total of 49 laboratories from the VRDL network across India were invited to participate in the pilot phase of the Pan-Dengue Molecular Serotyping External Quality Assessment Scheme (EQAS). During Round 1 (December 2024), 34 laboratories enrolled in the programme and 31 laboratories submitted results within the stipulated timeline, corresponding to a response rate of 91.2%. To further expand participation, Round 1 Phase-II (July 2025) was conducted, during which 40 laboratories enrolled and 38 laboratories submitted results, resulting in a response rate of 95.0%. In Round 2 (February 2026), the programme was expanded to include 71 invited laboratories. A total of 56 laboratories enrolled and 53 laboratories submitted results, yielding a response rate of 94.6%. Participating laboratories represented multiple States and Union Territories across India, including Andhra Pradesh, Assam, Bihar, Chandigarh, Chhattisgarh, Gujarat, Haryana, Jammu & Kashmir, Jharkhand, Karnataka, Kerala, Madhya Pradesh, Maharashtra, Manipur, New Delhi, Odisha, Puducherry, Punjab, Rajasthan, Sikkim, Tamil Nadu, Telangana, Tripura, Uttarakhand, Uttar Pradesh, and West Bengal (**Figure 1**).

### Homogeneity and stability assessment of EQAS/PT panels

#### Homogeneity assessment

The homogeneity of the lyophilized Dengue molecular serotype proficiency testing (PT) panels was evaluated in accordance with ISO 13528 recommendations using real-time RT-PCR cycle threshold (Ct) values as the assigned measurement parameter. Six randomly selected vials from the production batch were analyzed under repeatability conditions following reconstitution. The standard deviation for proficiency assessment (SDPA) was established at 2.0 Ct, while the between-sample standard deviation (Ss) was calculated as 0.42 Ct. The observed Ss value was below the acceptance criterion of 0.3 × SDPA (0.60 Ct), demonstrating satisfactory between-vial homogeneity (**Table 1**). The mean Ct values obtained from randomly selected vials showed minimal variation, indicating uniform distribution of target RNA within the lyophilized serum matrix. These findings confirmed that the PT items were sufficiently homogeneous for use in external quality assessment of dengue molecular diagnostics.

**Table 1:** Homogeneity assessment (based on RT-PCR Ct values).

Sl. No.	Standard deviation for proficiency assessment (SDPA, Ct)	Between-samples standard deviation, Ss (Ct)	Homogeneity check (Ss ≤ 0.3 × SDPA)	Result
1	2.0 Ct	0.42 Ct	0.42 ≤ 0.60	Homogeneous

#### Short-term stability during transportation

**Table 2** reveals the stability of the lyophilized PT panels under simulated transportation conditions was evaluated at room temperature (20-35 °C) for up to 240 hours (10 days). The mean Ct value obtained at each time point was compared with the baseline value measured immediately after production.

**Table 2:** Stability assessment during transportation (lyophilized serum at room temperature) Reference Mean Ct (Initial) = 28.3.

Sl. No.	Time period (Room temperature)	SDPA (Ct)	ABS (x̄ - ȳ)	Stability check ABS (x̄ - ȳ) ≤ 0.3 × SDPA	Result
1	24 Hours	2.0	0.08	0.08 ≤ 0.60	Stable
2	72 Hours	2.0	0.15	0.15 ≤ 0.60	Stable
3	120 Hours	2.0	0.24	0.24 ≤ 0.60	Stable
4	168 Hours (7 Days)	2.0	0.38	0.38 ≤ 0.60	Stable
5	240 Hours (10 Days)	2.0	0.52	0.52 ≤ 0.60	Stable

The absolute difference between the mean Ct values of stored and reference samples ranged from 0.08 Ct at 24 hours to 0.52 Ct at 240 hours (10 days). All observed differences remained below the predefined acceptance limit of 0.60 Ct (0.3 × SDPA). These results consistently indicated that the lyophilized PT materials retained molecular integrity during simulated transportation and remained stable for at least 10 days at ambient temperature.

#### Long-term stability during storage at 2-8 °C

Long-term stability studies were performed using lyophilized PT panels stored at 2-8 °C and evaluated at 1, 3, and 6 months (**Table 3**). The absolute differences between baseline and stored samples were 0.10 Ct, 0.21 Ct, and 0.34 Ct, respectively. All measured differences were substantially below the acceptance threshold of 0.60 Ct, indicating no significant degradation of viral RNA during refrigerated storage. The results support a shelf life of at least six months for the lyophilized Dengue molecular PT panels when stored under recommended conditions.

**Table 3:** Stability assessment during storage at 2-8 °C (lyophilized material).

Sl. No.	Time period	SDPA (Ct)	ABS (Difference in mean Ct)	Stability check ABS ≤ 0.3 × SDPA	Result
1	1 Month	2.0	0.10 Ct	0.10 ≤ 0.60	Stable
2	3 Months	2.0	0.21 Ct	0.21 ≤ 0.60	Stable
3	6 Months	2.0	0.34 Ct	0.34 ≤ 0.60	Stable

#### Stability following reconstitution

To evaluate post-reconstitution stability, PT materials were reconstituted with nuclease free molecular grade water and stored at −20 °C. Samples were tested after 7, 14, and 30 days of storage (**Table 4**). The absolute differences from baseline Ct values were 0.05 Ct, 0.11 Ct, and 0.19 Ct at 7, 14, and 30 days, respectively. All values remained within the acceptance criterion of 0.60 Ct, confirming that reconstituted PT materials remained stable for at least 30 days when stored at −20 °C.

**Table 4:** Stability assessment after reconstitution and storage at -20 °C.

Sl. No.	Time period	SDPA (Ct)	ABS (Difference in mean Ct)	Stability check ABS ≤ 0.3 × SDPA	Result
1	7 Days	2	0.05 Ct	0.05 ≤ 0.60	Stable
2	14 Days	2	0.11 Ct	0.11 ≤ 0.60	Stable
3	30 Days	2	0.19 Ct	0.19 ≤ 0.60	Stable

Overall the homogeneity and stability studies provided consistent evidence that the lyophilized serum-based Dengue molecular serotype PT panels fulfilled the acceptance criteria specified in ISO 13528 for proficiency testing materials. The observed variability in RT-PCR Ct values was minimal across production vials and throughout the evaluated storage and transportation conditions. These findings support the suitability of the developed PT panels for use in external quality assessment programs aimed at evaluating laboratory performance in dengue virus molecular detection and serotype identification.

### Panel distribution and testing

**Figure 2:**
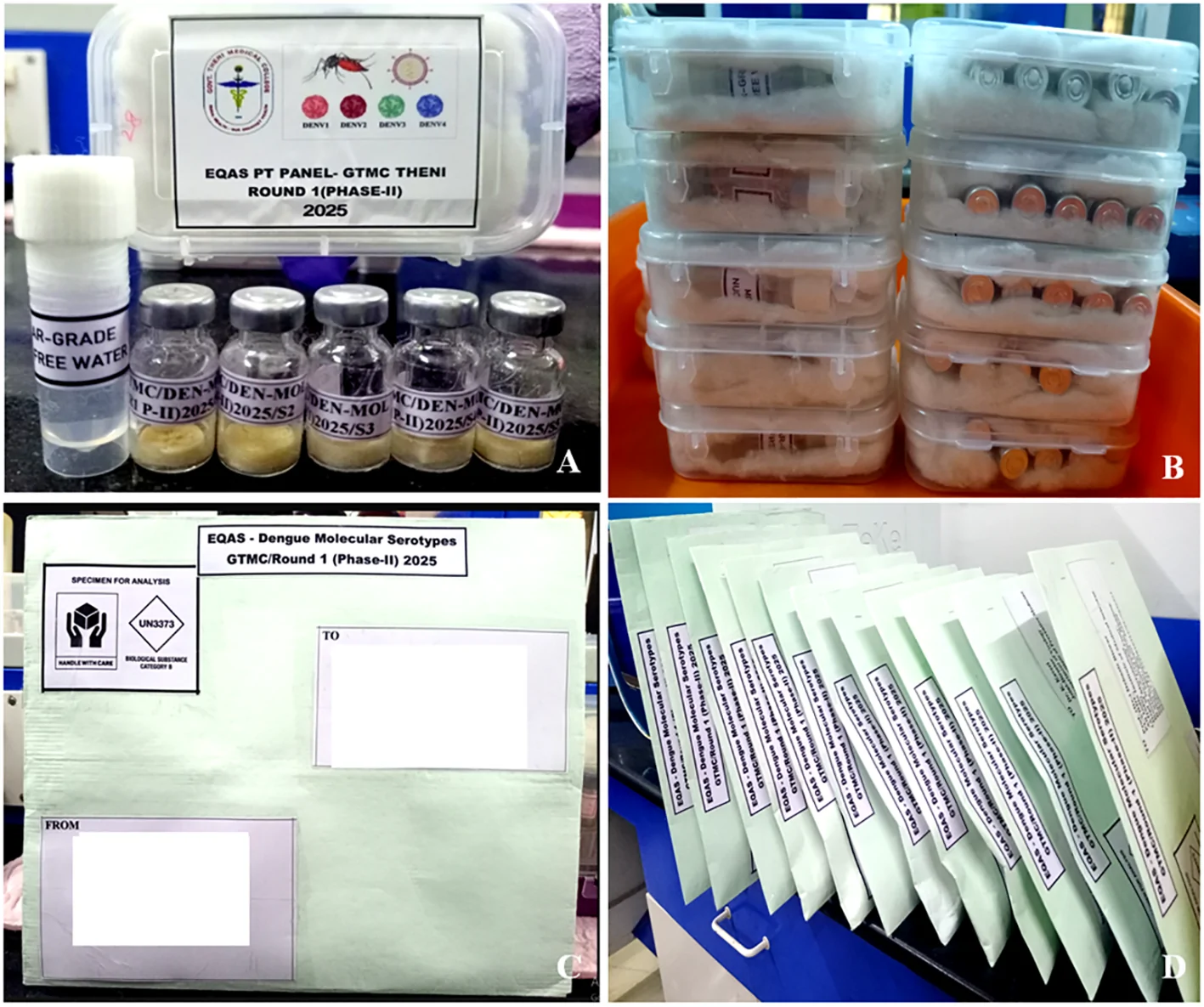
Preparation, packaging, and distribution of the dengue molecular serotypes external quality assessment scheme (EQAS) proficiency testing panel (Round 1, Phase II, 2025). **(A)** EQAS PT panel comprising lyophilized serum samples, along with molecular-grade nuclease-free water. **(B)** Secondary packaging containing individually packed lyophilized PT panels arranged for ambient (room-temperature) shipment. **(C)** Outer shipping package labeled in accordance with biological substance transport regulations, displaying sender and recipient information. **(D)** Individually packed labeled EQAS panels for distribution to participating laboratories.

Each PT panel consisted of five coded lyophilized serum samples (500 µL each) containing dengue virus-positive and dengue virus-negative specimens (**Figure 2**). The lyophilized serum samples were designed for stable transport at room temperature, eliminating the need for cold-chain logistics. The panels were distributed to participating laboratories along with instructions for reconstitution, testing, and reporting through the NIE- PT Panel Portal. All participating laboratories tested the samples using their routine molecular diagnostic workflow and reported qualitative results, serotype identification, and Ct values.

### Laboratory performance

The overall laboratory performance (**Table 5**) was assessed based on concordance between participant-reported results and assigned reference results. During Round 1 (December 2024), all 31 reporting laboratories correctly identified all PT samples and achieved 100% concordance. Similarly, during Round 1 Phase-II (July 2025), all 38 reporting laboratories achieved 100% concordance, demonstrating excellent performance in dengue virus detection and serotype identification. In Round 2 (February 2026), among the 53 laboratories that submitted results, 50 laboratories correctly identified all five PT samples and achieved 100% concordance (5/5 correct results). Two laboratories achieved 80% concordance (4/5 correct results), while one laboratory achieved 60% concordance (3/5 correct results).

**Table 5:** Performance of participating laboratories.

Performance category	Round 1	Round 1 phase-II	Round 2
100% Concordance	31 (100%)	38 (100%)	50 (94.3%)
80% Concordance	0	0	2 (3.8%)
60% Concordance	0	0	1 (1.9%)
Total Reporting Laboratories	31	38	53

### Molecular assays and diagnostic kits used

**Figure 3:**
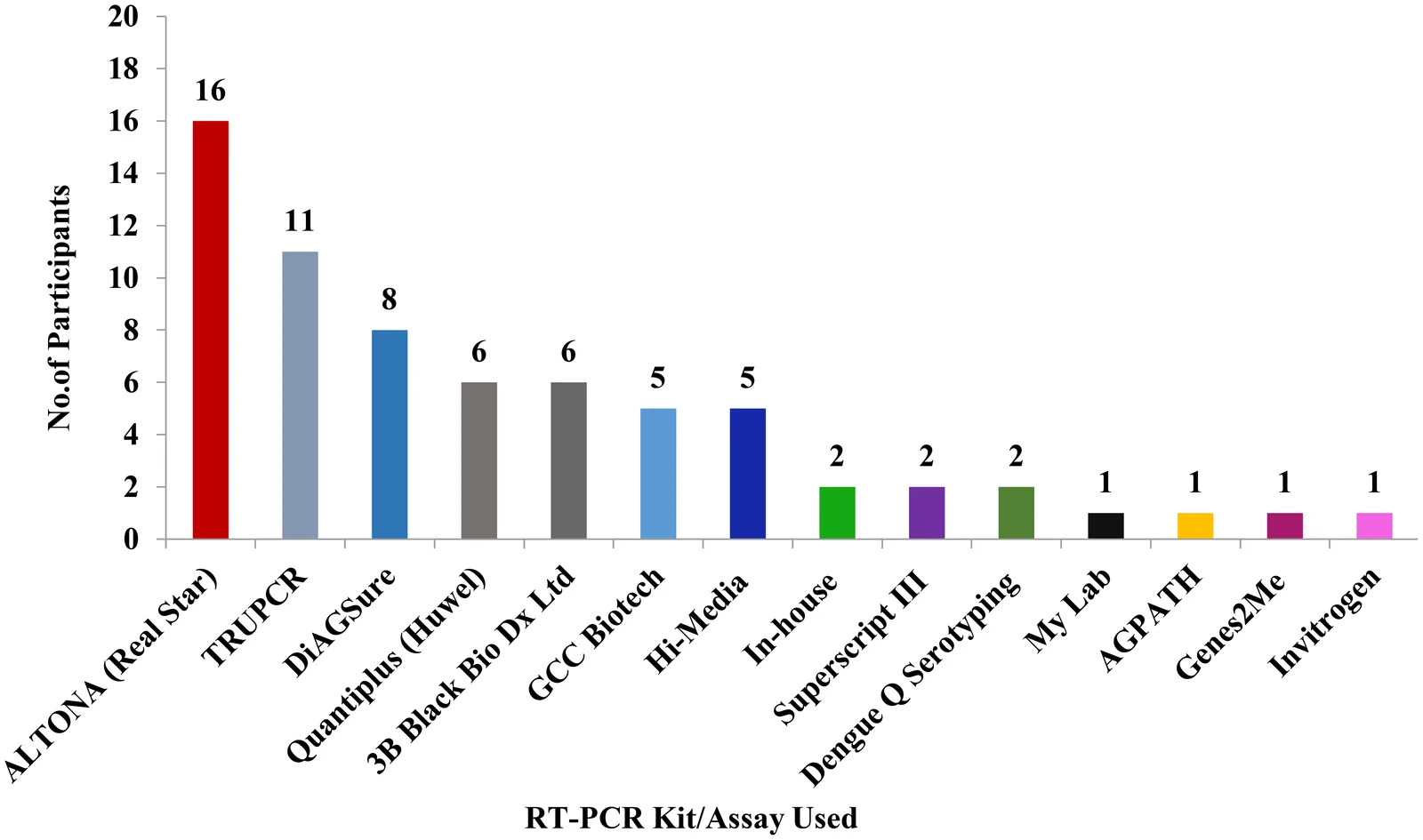
Distribution of molecular diagnostic kits used by participating laboratories during pan-dengue molecular serotyping EQAS (December 2024-February 2026).

A variety of commercial real-time RT-PCR kits were utilized by participating laboratories for dengue virus detection and serotype identification. The most frequently used assay kit across all EQAS rounds was the RealStar^®^ Dengue RT-PCR Kit (Altona Diagnostics), followed by the TRUPCR^®^ Dengue Real-Time RT-PCR Kit. Other commonly used platforms included the DiAGSure Dengue Multiplex RT-PCR Kit, Quantiplus Dengue RT-PCR Serotyping Kit (Huwel), 3B Black Bio Dx Ltd Dengue RT-PCR Kit, and several institution-specific validated assays (**Figure 3**). The distribution of assay usage highlighted the diversity of molecular diagnostic platforms employed within the VRDL network laboratories. Despite the use of different extraction methods, RT-PCR kits, and instrument platforms, a high level of concordance was observed across participating laboratories, indicating the robustness and reliability of the molecular diagnostic systems used for dengue virus detection and serotyping.

### Concordance analysis

As shown in **Table 6**, all laboratories participating in Round 1 and Round 1 Phase-II correctly identified the expected dengue virus detection and serotype results, resulting in an overall concordance of 100%. During Round 2, the overall concordance remained high, with 94.3% (50/53) laboratories achieving complete agreement with the reference results. Three laboratories reported discordant results, resulting in reduced concordance scores of 80% and 60%. According to the predefined EQAS acceptance criterion of ≥ 80% concordance (i.e. 4/5 marks), 52 of the 53 reporting laboratories (98.1%) successfully passed the proficiency testing programme during Round 2.

**Table 6:** Overall EQAS performance summary.

EQAS round	Reporting laboratories	Laboratories passing (≥80%)	Pass (%)
Round 1 (Dec 2024)	31	31	100
Round 1 Phase-II (Jul 2025)	38	38	100
Round 2 (Feb 2026)	53	52	98.1
Overall	122	121	99.3

### Discordant results

A small number of discordant results were observed during Round 2. The discrepancies included incorrect detection and/or serotype assignment of PT panel samples. Laboratories reporting discordant results were contacted for further investigation and troubleshooting. Root-cause analysis indicated possible issues related to RNA extraction efficiency, assay performance, result interpretation, or reporting errors. Investigation showed that both the laboratories tested the PT panel after the validated stability period and submitted results approximately 15 days after the reporting deadline. No deficiencies related to PT panel preparation, transportation, or reporting were identified. The retained aliquots of the PT samples were subsequently retested at the reference laboratory, confirming the expected dengue virus serotypes. These findings indicate that the discordance was not due to nucleic acid degradation or other pre-analytical factors but was most likely attributable to delayed testing beyond the validated stability period.

### Feedback and corrective actions

Individual performance reports were generated and communicated to all participating laboratories through the NIE PT Panel Portal. Laboratories with discordant results were advised to undertake corrective and preventive actions (CAPA), including review of extraction procedures, assay performance, instrument maintenance, and staff training. Follow-up communication was conducted to ensure implementation of corrective measures and improvement in laboratory performance.

### Participation trends in the dengue EQAS program

**Figure 4:**
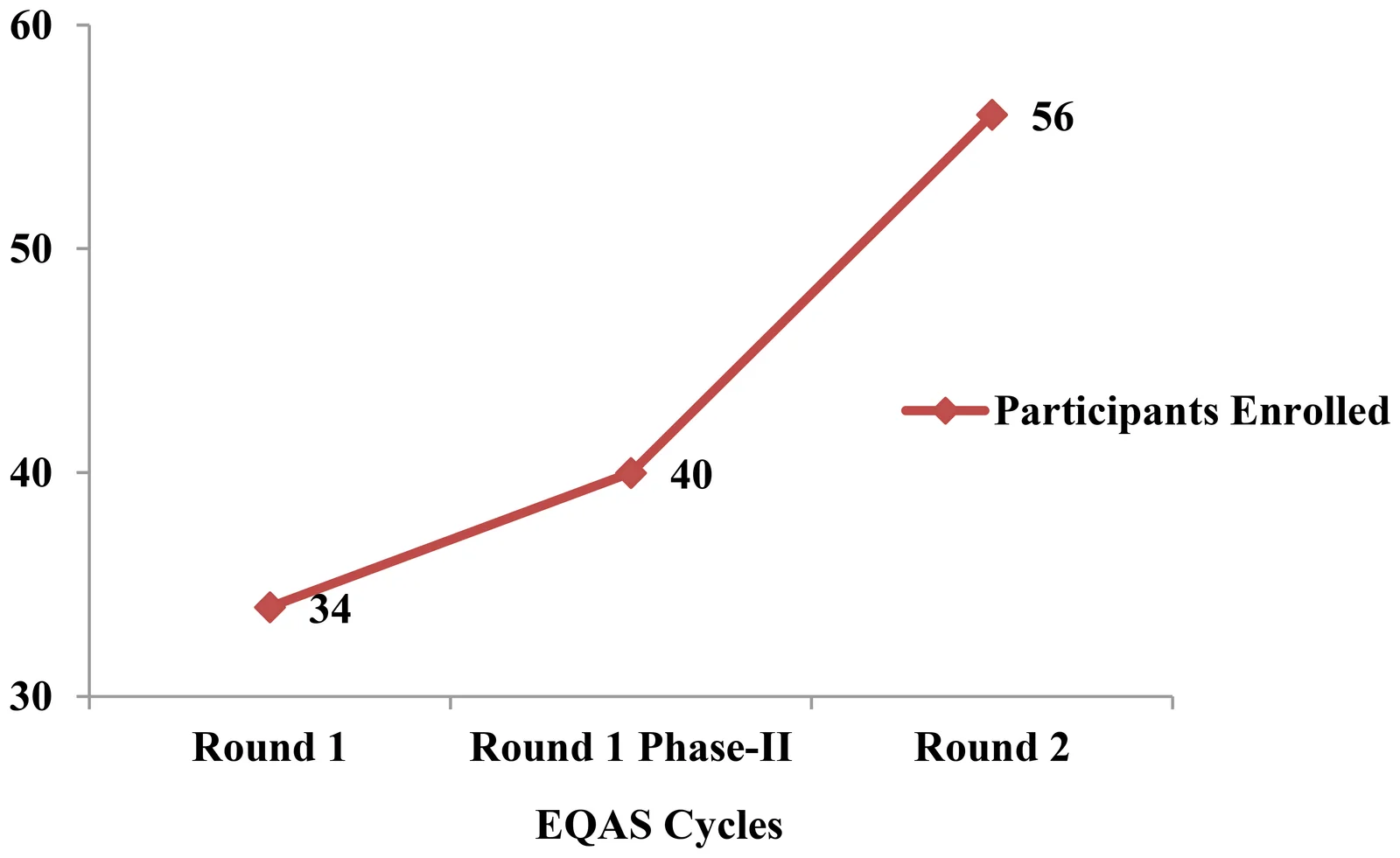
Trend in laboratory enrollment across Dengue EQAS rounds.

A line chart (**Figure 4**) denotes a steady increase in laboratory participation was observed throughout the implementation of the Dengue External Quality Assessment Scheme (EQAS). The number of enrolled laboratories increased from 34 in Round 1 to 40 in Round 1 Phase-II, reflecting a 17.6% growth. Subsequently, participation reached 56 laboratories in Round 2, representing a 40.0% increase over Round 1 Phase-II. Overall, laboratory enrollment increased by 64.7% between Round 1 and Round 2, indicating expanding engagement and acceptance of the EQAS program.

## Discussion

The present study describes the development and implementation of a nationwide Pan-Dengue Molecular Serotyping EQAS for evaluating the proficiency of participating laboratories in dengue virus detection and serotype identification. The programme was successfully conducted across three rounds between December 2024 and February 2026 and achieved a consistently high level of performance among participating VRDLs in India. The dispatched PT panels were homogeneous and stable after assessment with acceptance criteria in ISO 13528 for proficiency testing materials (Zhang et al., 2023; Zhang et al., 2024; Nguyen et al., 2025). These findings emphasize the importance of external quality assessment as a key component of laboratory quality management systems and its contribution to strengthening molecular diagnostic services and surveillance activities in India (Sciacovelli et al., 2006; Miller et al., 2011; Carey et al., 2018).

A progressive increase in laboratory participation was observed during the study period, with the number of enrolled laboratories increasing from 34 in Round 1 to 56 in Round 2. This expansion reflects the growing awareness of quality assurance requirements and the increasing capacity of laboratories within the national viral diagnostic network. The consistently high response rates observed across all EQAS rounds indicate strong engagement and commitment among participating laboratories towards maintaining quality standards and ensuring reliable molecular testing. Similar findings have been reported in other proficiency testing programmes, where regular participation has been associated with improved laboratory performance and enhanced standardization of diagnostic practices [32, 33, & 40].

The overall performance of participating laboratories was highly satisfactory. All reporting laboratories achieved 100% concordance during Round 1 and Round 1 Phase-II, while 94.3% of laboratories achieved complete concordance during Round 2. The few discordant results observed during Round 2 may be attributed to variations in nucleic acid extraction efficiency, assay performance, instrument calibration, sample handling, or interpretation of results. Nevertheless, the overall concordance rate remained exceptionally high, demonstrating the robustness of molecular testing practices across the participating laboratories. These findings indicate that the quality assurance measures, training activities, and standardized testing protocols implemented within the VRDL network have contributed significantly to strengthening laboratory performance (Deshpande et al., 2023; Ratchagadasse et al., 2026).

External quality assessment programmes provide an objective mechanism for evaluating laboratory performance and identifying analytical deficiencies that may not be detected through routine internal quality control procedures (Miller et al., 2011; Carey et al., 2018). Molecular diagnostic assays involve multiple analytical steps, including sample preparation, RNA extraction, amplification, detection, and result interpretation. Errors occurring at any stage may influence test outcomes and compromise diagnostic accuracy. In the present study, laboratories reporting discordant results were subjected to root-cause analysis and provided with appropriate corrective and preventive actions (CAPA). This highlights the role of EQAS as a continuous quality improvement tool that not only assesses laboratory performance but also facilitates the identification and correction of potential weaknesses within laboratory processes (Sciacovelli et al., 2006; Plebani, 2012).

One of the major strengths of the present programme was the use of lyophilized serum samples (500 µL) for proficiency testing. Lyophilized samples offer several operational advantages, including enhanced stability, ease of storage, reduced dependence on cold-chain logistics, and suitability for transportation over long distances. The successful distribution of PT panels to laboratories across multiple states and union territories without compromising sample integrity demonstrates the feasibility of using lyophilized materials for large-scale molecular EQAS programmes. Homogeneity and stability testing confirmed the reliability of the panels under different storage conditions, supporting their use for future national and regional quality assessment initiatives (Carey et al., 2018; Bartulović et al., 2025).

An important feature of this EQAS was the inclusion of dengue virus serotype identification in addition to routine viral RNA detection. Molecular serotyping plays a crucial role in dengue surveillance by enabling monitoring of circulating serotypes, identification of serotype replacement events, and assessment of outbreak dynamics (Deshpande et al., 2023; Dieng et al., 2024; Paz-Bailey et al., 2024; Malik et al., 2025; Sani et al., 2025). Accurate serotype determination contributes to improved epidemiological investigations and supports public health interventions aimed at dengue prevention and control. The high concordance observed for serotype identification among participating laboratories indicates the technical competence of the laboratories and the reliability of the molecular platforms currently employed within the network.

The use of the National Institute of Epidemiology (NIE) Portal further strengthened the EQAS process by providing a centralized platform for participant registration, result submission, data management, performance evaluation, and dissemination of feedback reports. Electronic reporting systems facilitate timely analysis of laboratory performance, improve traceability of data, and support documentation requirements for quality management systems and accreditation standards such as ISO 15189 (Tembo et al., 2026). The integration of digital platforms into EQAS activities represents an important step towards modernization and sustainability of laboratory quality assurance programmes.

The findings of this study are also supported by experiences from other arbovirus proficiency testing programmes, including those for Zika virus, Chikungunya virus, Yellow Fever virus, and West Nile virus. These programmes have consistently demonstrated that regular participation in EQA schemes strengthens diagnostic capacity, improves inter-laboratory comparability, enhances staff competency, and supports public health surveillance systems. Beyond performance assessment, such programmes contribute to harmonization of laboratory practices, early identification of technical deficiencies, and development of corrective actions. The increasing participation and excellent performance observed in the present Dengue Molecular Serotyping EQAS suggest that the VRDL network is well-positioned to support reliable molecular surveillance and outbreak preparedness activities across India (Deshpande et al., 2023).

Although the results of the present study are highly encouraging, certain limitations should be acknowledged. The PT panels consisted of a limited number of samples and may not fully represent the diversity of viral loads and serotypes encountered during routine clinical testing. Furthermore, laboratory performance was evaluated under controlled proficiency testing conditions, which may differ from routine diagnostic settings. Future EQAS rounds may benefit from the inclusion of low-positive samples, mixed-serotype specimens, and samples with varying viral loads to further challenge laboratory performance and assess analytical sensitivity under more complex conditions.

## Conclusion

The Pan-Dengue Molecular Serotyping EQAS highlighted excellent laboratory performance and a high level of proficiency among participating VRDLs laboratories across India. The consistently high concordance rates observed throughout the study confirm the effectiveness of the quality assurance framework established within the national laboratory network. The programme successfully strengthened molecular diagnostic quality, enhanced confidence in dengue surveillance data, and provided valuable opportunities for continuous quality improvement. Continued implementation and expansion of such EQAS initiatives will play a critical role in maintaining high standards of molecular diagnostics, supporting dengue surveillance programmes, and strengthening preparedness for emerging and re-emerging viral diseases in India.

## Data Availability

The original contributions presented in the study are included in the article/**Supplementary Material**, further inquiries can be directed to the corresponding author/s.
