# Supplementary material for: External quality assurance of molecular serotype detection of dengue virus RNA among virus research and diagnostic laboratories in India
Source: Front Cell Infect Microbiol. 2026 Sep 3;16:1919409. doi: 10.3389/fcimb.2026.1919409 (PMC13581684; doi:10.3389/fcimb.2026.1919409)
Supplement: Supplementary file 1 [file SupplementaryFile1.docx]

**Figure S1:**

Fig S1: Workflow of the Pan-Dengue Molecular Serotyping EQAS Program
